# Why do family doctors prescribe potentially inappropriate medication to elderly patients?

**DOI:** 10.1186/s12875-016-0482-3

**Published:** 2016-07-22

**Authors:** Karen Voigt, Mandy Gottschall, Juliane Köberlein-Neu, Jeannine Schübel, Nadine Quint, Antje Bergmann

**Affiliations:** Department of General Practice/Medical Clinic III, Medical School, Technische Universität Dresden, Dresden, Germany; Department of Health Care Management and Public Health, Schumpeter School of Business and Economics, University of Wuppertal, Wuppertal, Germany

**Keywords:** Potentially inappropriate medication, Family doctor, Polypharmacy, Mixed methods

## Abstract

**Background:**

Based on changes in pharmacokinetics and –dynamics in elderly patients, there are potentially inappropriate medications (PIM) that should be avoided in patients aged ≥ 65 years. Current studies showed prescription rates of PIM between 22.5 and 28.4 % in the primary care setting. The evidence concerning reasons for PIM prescription by FPs is limited.

**Methods:**

This mixed method study consisted of three research parts: 1) semi-standardized content analysis of patients’ records, 2) qualitative interviews with FPs using a) open questions and b) selected patient-specific case vignettes and 3) qualitative interviews with FPs’ medical assistants. The integration of qualitative interviews was used to explain the quantitative results (triangulation design). PIM were identified according to the German PRISCUS list. Descriptive and multivariate statistical analysis was done using SPSS 22.0. Qualitative content analysis of interviews was used to classify the content of the interviews for indicating pertinent categories. All data were pseudonymously recorded and analyzed.

**Results:**

Content analysis of 1846 patients’ records and interviews with 7 related FPs were conducted. Elderly patients [*n* = 1241, mean age: 76, females: 56.6 %] were characterized in average by 8.3 documented chronic diagnosis. 23.9 % of elderly patients received at least one PIM prescription. Sedatives/hypnotics were the most frequent prescribed PIM-drugs (13.7 %). Mental disorders, gender and number of long-term medication were detected as predictors for the probability of a PIM prescription. Common reported reasons for PIM prescription by FPs concerned limited knowledge regarding PIM, limited applicability of PIM lists in daily practice, lack of time, having no alternatives in medication, stronger patient-related factors than age that influence prescription, own bad experiences regarding changes of medication or refusal of following prescriptions of sedative/hypnotics.

**Conclusions:**

It is essential to see FPs in a complex decision making situation with several influencing factors on their prescribing, including: patient-oriented prioritization, FPs’ experiences in daily practice, FPs’ knowledge regarding existing recommendations and their trust in it and organizational characteristics of FPs’ daily medical practice. These pros and cons of PIM prescription in elderly patients should be considered in FPs’ advanced training.

## Background

The discussion of challenges in medical care for elderly patients (65 years and older) is focused on multimorbidity and polypharmacy. Regarding age-related changes in pharmacokinetics and -dynamics, the elderly patients’ renal metabolism and clearance as well as first-pass effect in the liver are delayed in time. This causes different actions of drug in geriatric vs. younger patients because the pharmaceutical drugs remain longer in the metabolic system of the elderly [1].

Prescription of medical drugs in geriatric patients has to consider these effects. But mostly there is no evidence regarding effect of drugs in the elderly. Clinical trials for testing efficacy of drugs often exclude older patients based on an upper-age limit, comorbid disease, reduced life expectancy, physical or mental impairments or use of drugs aiming to minimize biases on outcomes of the study. But the results of efficacy studies that exclude older patients do rarely fit patients in daily medical practice [2, 3].

Common empirical data show higher hospital admission rates in elderly patients because of adverse drug reactions or drug-drug reactions [4–6]. Regarding this and the complexity of problems with medication for the elderly, expert groups in several countries have developed so called black lists of drugs for elderly patients, describing potentially inappropriate medication (PIM).

Due to different formalities and drug markets, country specific PIM lists are necessary [7]. These country-specific lists include drugs that are associated with higher risks of intolerance, adverse drug reaction/events or drug-disease interactions in elderly patients [8–14].

In Germany the first black list ‘PRISCUS’ (PRerequISites for a new health Care model for elderly people with mUltiple morbiditieS) was consensually developed in 2010 by an expert group of geriatrics, pharmacologists and family practitioners. PRISCUS currently includes 83 drugs that should be avoided or prescribed at a lower dose in elderly patients. These recommendations are listed with expert statements offering alternatives. In case of unavoidability of PIM prescriptions, recommendations for drug monitoring are given [15].

Rates of PIM prescriptions range worldwide between 12 and 65 % depending on the setting and the used screening tool. A systematic review determined a median PIM prescription rate of 20.5 % (interquartile range 18.1 to 25.6 %) in the primary care setting between 1950 and 2011. The review included 19 studies of 11 different countries that examined PIM almost by Beers criteria [16]. Current studies examining older patient samples (mean age > 80 years) showed prescription rates between 22.5 and 28.4 % in the primary care setting [17, 18].

Since these rates seem to be high, it would be useful to understand the prescribing of PIM by family practitioners (FP) to generate feasible and need-oriented solutions for improvement of medical care in the elderly. In this context, more evidence is needed regarding FPs’ knowledge of PIM as well as associated recommendations and their acceptance and implementation in daily practice.

There is evidence that polypharmacy increases the risk of PIM in elderly patients [6, 18, 19]. Furthermore, there are indications that FPs’ knowledge of prescribing for the elderly is partly insufficient (more frequently in older FPs) whereas they report a high self-rated confidence in prescribing [20]. Reported main barriers for appropriate prescribing seem to be related to FPs’ lack of education regarding PIM, to organizational characteristics (i.e. lack of time, limited answer options on insurance formularies, communication difficulties with other doctors) and to patients (large number of medications, unknown medications, costs). These findings are based on two smaller studies using a validated survey instrument to examine FPs’ knowledge of prescribing for the elderly. Appropriateness was assessed by standardized case vignettes [20, 21].

Based on the limited knowledge concerning reasons for PIM prescription by FPs we examined reasons for PIM prescription a) in general and b) using real individual case vignettes of FPs that were detected beforehand through record analysis. Aims of this analysis were

1. to give an overview of rates of PIM prescription in our study sample of elderly multimorbid patients with polymedication in the outpatient primary care setting,
2. to explain influencing factors on prescription of PIM,
3. to examine knowledge and application of PRISCUS and
4. to understand FPs’ reasons for prescription of PIM.

## Methods

Our study was coordinated at the Department of General Practice of the Technische Universität (TU) Dresden and took place from March 2013 to June 2014. The study was conducted in cooperation with the Department of Health Care Management and Public Health/Schumpeter School of Business and Economics at the University of Wuppertal.

### Study design

The study was designed as a mixed methods study consisting of three research parts:

1. semi-standardized content analysis of patients’ records regarding morbidity, drug prescriptions, admissions/referrals to specialist,
2. qualitative interviews with FPs that focused on challenges and barriers in daily medical care of patients with multimorbidity and polypharmacy using a) open questions to explore these issues in general and b) selected patient-specific case vignettes for each FP based on results of [1] regarding comparable topics (PIM: prescription of hypnotics; medication change; intersectoral cooperation with in- and out-patient specialists), and
3. qualitative interviews with their medical assistants to explore their point of view regarding organization-related barriers, challenges and room for improvement for medical care of patients with multimorbidity and polypharmacy [22].

Results of qualitative interviews with FP and their medical assistants were used to help explain the quantitative results (triangulation design [23]). More detailed information on our study design, setting and research instruments are already published elsewhere [24].

This publication focuses on results of subanalysis of research parts (1) and (2) regarding the prescription of PIM by FPs.

### Inclusion of study participants

All 33 FPs from academic teaching practices of the Department of General Practice of TU Dresden situated in Dresden were invited for participation in the study. After confirmation of participation by the FPs, all chronic patients of each participating practice were invited for participation if they met following inclusion criteria: a) at least two parallel chronic diseases and b) two prescribed long-term medications at least 6 months before 2012. Furthermore, all medical assistants of the participating FPs were asked to participate in qualitative interviews.

### Data collection

Patients’ records [1] were analyzed from March 2013 to May 2014 by our project assistants at FPs’ practices using a semi-standardized entry mask in SPSS 22.0. Patient records of a randomized quarter of the year 2012 were retrospectively analyzed per patient, and information regarding morbidity, admissions/referrals to specialists and non-drug and drug prescriptions (including documented adverse events) were documented. Prescriptions by specialists were only included if FPs had documented them in the patient records based on the findings reports sent by the specialists or direct information by the patients.

The quarters were assigned randomly to each patient. Qualitative face-to-face interviews [2] were conducted by 2 experienced interviewers (scientific project staff) with 7 FPs around 2–3 weeks after finishing record analysis. Interviews took place in FPs’ practices and each interview lasted between 40 and 60 min. Confronting FPs with their real-life decisions in individual case vignettes that were chosen from analyzed patients’ records (see Table 1) we asked them

**Table 1:** Description of case vignettes regarding documented prescription of PIM

FP	Documented chronic conditions of the case (ICD-10)	Documented acutediagnosis of the case (ICD-10)	Prescription of PIM per quarter: agents/amount (prescribed daily dosage)	Further agents for long-term treatment/same quarter
FP1	D25: Leiomyoma of uterus	F43: Reaction to severe stress, and adjustment disorders	Zolpidem/80 pellets (missing)	Candesartan, Carbamazepine, Doxepin, Gabapentin, Metoprolol, Metformin, Pravastatin
	E22: Hyperfunction of pituitary gland			
	E78: Disorders of lipoprotein metabolism			
	G25: Other extrapyramidal and movement disorders			
	G47: Sleep disorders			
	G56: Mononeuropathies of upper limb			
	G58: Other mononeuropathies			
	I10: Essential (primary) hypertension			
	I65: Occlusion and stenosis of precerebral arteries			
	R55: Syncope and collapse			
FP2	F13: Mental and behavioral disorders due to use of sedatives or hypnotics	M54: Dorsalgia	Zopiclone/60 pellets (7.5 mg/day), Lorazepam/missing (2.5 mg/day)	Acetylsalicylic acid, Amlodipine, Calcitrol, Carvedilol, Cholecalciferol, Minoxidil, Mirtazapine, Pantoprazole, Torasemide, Valsartan
	F34: Persistent mood [affective] disorders			
	F40: Phobic anxiety disorders G47: Sleep disorders			
	I10: Essential (primary) hypertension			
	N18: Chronic kidney disease			
	T88: Other complications of surgical and medical care, not elsewhere classified			
FP3	E03: Hypothyroidism	J06: Acute upper respiratory infections of multiple and unspecified sites	Zolpidem/80 pellets (10 mg/day)	Beclomethasone, Bisoprolol, Cobalamin, Duloxetine, Hydrochlorothiazide, Lactulose, Levothyroxine, Metoclopramide, Omeprazole, Salbutamol, Tramadol,
	E53: Deficiency of other B group vitamins			
	I35: Nonrheumatic aortic valve disorders I50: Heart failure			
	K29: Gastritis and duodenitis			
	M48: Other spondylopathies			
	Q61: Cystic kidney disease			
	R15: Faecal incontinence			
	R52: Pain, not elsewhere classified			
FP4	D86: Sarcoidosis	none	Zolpidem/100 pellets (10 mg/day)	Hydrochlorothiazide, Naloxone, Nitrendipine, Pregabalin, Tilidine, Valsartan
	E78: Disorders of lipoprotein metabolism and other lipidaemias			
	G47: Sleep disorders			
	H53: Visual disturbances			
	I10: Essential (primary) hypertension			
	I83: Varicose veins of lower extremities			
	M25: Other joint disorders, not elsewhere classified			
	M81: Osteoporosis without pathological fracture			
	N17: Acute renal failure			
	R26: Abnormalities of gait and mobility			
	Z93: Artificial opening status			
FP5	C80: Malignant neoplasm, without specification of site	R07: Pain in throat and chest	Zopiclone/missing (7.5 mg/day)	Bisoprolol, Hydrochlorothiazide, Ramipril, Trimipramine, Pantoprazole, Acetylsali-cylic acid, Ibuprofen
	H53: Visual disturbances			
	I10: Essential (primary) hypertension			
	I27: Other pulmonary heart diseases			
	I34: Atrial fibrillation and flutter			
	K43: Ventral hernia			
	R26: Abnormalities of gait and mobility			
	Z93: Artificial opening status			
	Z96: Presence of other functional implants			
FP6	E11: Type 2 diabetes mellitus	none	Zopiclon/120 pellets (missing)	Allopurinol, Amlodipine, Enalapril, Moxonidine
	I10: Essential (primary) hypertension			
	I50: Heart failure			
	M10: Gout			
	N08: Glomerular disorders in diseases classified elsewhere			
	N18: Chronic kidney disease			
FP7	E78: Disorders of lipoprotein metabolism and other lipidaemias	M54: Dorsalgia	Zopiclon/180 pellets (7.5 mg/day)	Allopurinol, Felodipine, Metoprolol, Olmesartan
	E79: Disorders of purine and pyrimidine metabolism			
	F51: Nonorganic sleep disorders I10: Essential (primary) hypertension			
	J44: Other chronic obstructive pulmonary disease

- to explain their case-related decisions of the prescription of a PIM substance and influencing factors on it for the respective case vignette,
- to discuss reasons for prescription of PIM in contrast to known recommendations generally and
- to assess their knowledge, usage and acceptance of recommendations in daily practice (German PRISCUS-list).

We used these real-life case vignettes to get more authentic answers by FPs and as concrete starting points for reflection of the PIM-topic in general. One patient (>65 years) with the most prescription of benzodiazepine receptor agonists (so called Z-drugs) not corresponding to recommendations of PRISCUS per each FP was selected for the respective case vignette (see Table 1).

### Analysis

Statistical analysis of data based on record analysis [1] were done using SPSS 22.0 to describe characteristics of the sample of patients > 65 years and especially of the subsample affected by PIM prescription. Screening all documented prescribed drugs, PIM were identified according to the German PRISCUS list. Differences in distributions were examined using chi^2^-test. *T*-test for unpaired samples was conducted to examine differences of means between different groups. A logistic regression was conducted to identify predictors for PIM-prescription.

The procedure of qualitative content analysis of interviews with FPs [2] is based on Mayring [25]. Two researchers analyzed the interview manuscripts by using MAXQDA® software to categorize contextual factors and individual reasons for PIM prescription from FPs’ point of view. The contents of the interviews were classified inductively to indicate pertinent categories. The derived categories were discussed and consented in the research team.

All data were pseudonymously recorded and analyzed. For integrating results, interview data (e.g. reported motives of prescription) were triangulated with data based on patient records’ analysis (e.g. relative frequencies of PIM prescription).

## Results

### Description of participating family physicians

Out of 33 academic teaching physicians of the Department of General Practice of TU Dresden, working in Dresden, 7 (of which 5 were female) participated in this study. The age of the participants was distributed between 43 and 61 years, and professional experiences were reported between 7 and 22 years. Out of 7 FPs, 3 worked at group practices and 4 at single practices, At the FPs’ practices, between 2 and 6 medical assistants were employed.

Of all the 7 FPs’ patients a number of 2826 met the inclusion criteria (≥2 chronic conditions and ≥ 2 related long-term medication), and 1846 patients (65.3 %) gave their consent for participation in the study. The number of participating patients per FP varied between a minimum of 120 and a maximum of 455. 55 % were females, and the age distribution was between 23 and 100 years with a mean age of 69 (±11 SD) years. 69.4 % of the patients were > 65 years old who will be in focus of following analysis.

Based on the small sample of participating FPs, the results of the study are limited regarding representativeness of FPs and patients. To check for potential biases we compared our data with results of comparable studies (external validation, see below).

### Description of the sample: elderly patients

Elderly patients [*n* = 1241, mean age: 76 (±6 SD), females: 56.6 %] were characterized in average by 8.3 [(±3.7 SD] documented chronic diagnosis (ICD 10) that include a total of 543 different ICD-Codes (shortened to first figure).

Most prevalent diagnosis described typical medical conditions of the metabolic syndrome: for 84.0 % of patients > 65 years essential hypertension (I10), for 49.3 % lipometabolic disorders (E78) and for 39.3 % diabetes mellitus 2 (E11) was documented (see Table 2). In 16.1 % of cases there was a co-occurrence of these three diagnoses as a hint for prevalence of a metabolic syndrome.

**Table 2:** Top Ten of documented chronic and acute diagnosis in patients > 65 years (*n* = 1241)

Chronic diagnosis		Absolute frequency	Relative frequency	Acute diagnosis		Absolute frequency	Relative frequency
I10	Essential hypertension	1076	84.0	M54	Dorsalgia	38	5.4
E78	Dyslipidemia	632	49.3	J06	Acute upper respiratory infections	35	5.0
E11	Diabetes mellitus type 2	503	39.3	N39	Other disorders of urinary system	23	3.3
I25	Chronic ischaemic heart disease	422	32.9	R10	Abdominal and pelvic pain	22	3.1
M81	Osteoporosis	209	16.3	R21	Rash and other nonspecific skin eruption	22	3.1
I83	Varicosis of lower extremities	193	15.1	M53	Other dorsopathies, not elsewhere classified	21	3.0
I48	Atrial flutter/fibrillation	193	15.1	F43	Reaction to severe stress	21	3.0
Z92	long-term (current) use of anticoagulants	183	14.3	M25	Other joint disorders, not elsewhere classified	19	2.7
M17	Gonarthrosis	182	14.2	G47	Sleep disorders	15	2.1
E79	Disorders of purine and pyrimidine metabolism	163	12.7	F32	Depressive episode	14	2.0

65.5 % of the elderly patients were given 4 and more drugs in long term medications (M: 6.3 ± 3.3 SD). The number of prescribed drugs correlated not very strongly with the number of chronic diagnosis (*r* = 0.34/*p* ≤ 0.001).

Regarding the interpretation of documented chronic diagnoses the limitation of validity based on missing or unspecific diagnoses needs to be considered. Patients’ records consist on information about chronic diagnoses documented primary for billing purposes. Although billed drugs and medical services need to be confirmed by documenting associated diagnoses, mismatches were found in our data. An exemplary analysis regarding congruence of prescriptions of thyroid medications and associated thyroid diagnosis showed insufficiently documented thyroid diagnosis in 26.8 % of patients with diagnosed or treated thyroid diseases [26].

### Prescription of potentially inappropriate medication

Out of the 83 PIM in the German PRISCUS list (including inappropriate drugs and in some cases inadequately dosed drugs), 41 PIM were prescribed in our sample. Using the method of record analysis, our data include all of FPs’ documented drug prescriptions, incl. private prescriptions that are not covered by the statutory health insurance respectively over the counter medication. Prescriptions by specialists were only included if FP had been informed and documented it. Since our data are related to FP prescription, the real PIM-prescription in general will be underestimated.

**Fig. 1:**
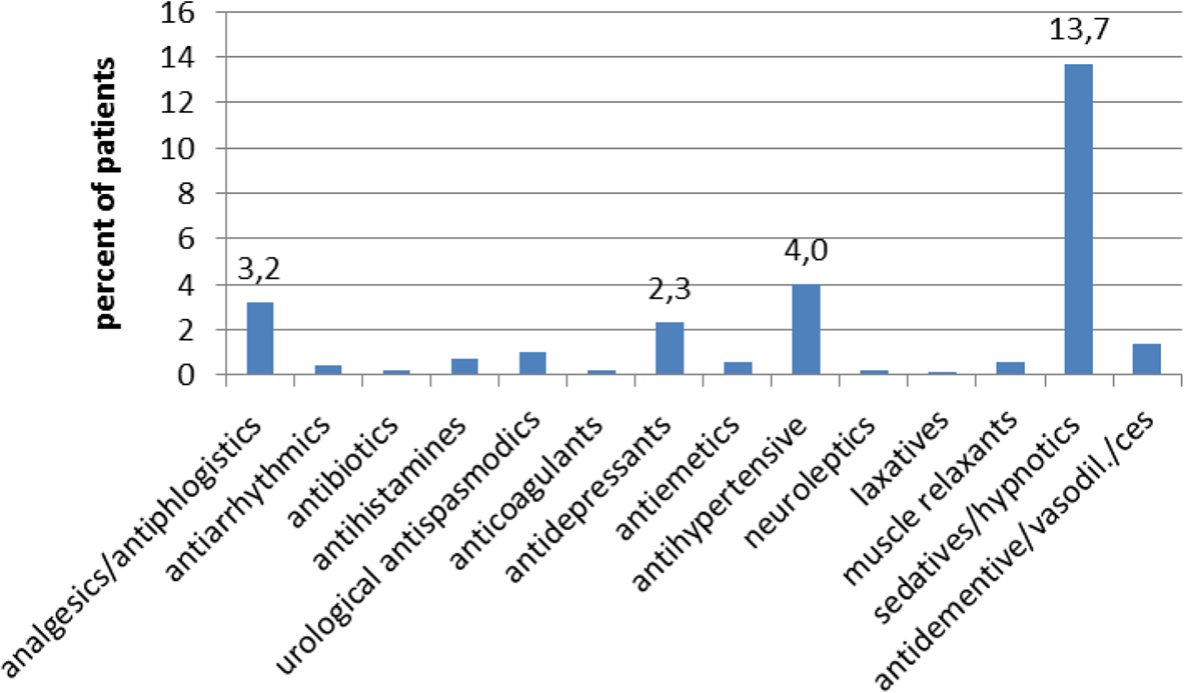
Frequencies of prescribed PIMs summarized in agents groups in elderly patients (*n* = 1241)

To summarize, 23.9 % of elderly patients received at least one PIM prescription, which is comparable to other studies reporting PIM prescription rates between 22.5 and 28.4 % of elderly patients in the primary care setting [17, 18]. 21.5 % of patients in our study got at least one PIM as medication for chronic conditions and 3.1 % to care for acute symptoms. Sedatives and hypnotics were the most frequent (13.7 %) prescribed PIM-drugs in patients > 65 years (see Fig. 1).

Zopiclon (4.3 %), Zolpidem (2.3 %), Diazepam and Doxacosin (each 1.9 %) were the top-3-PIM in our sample (see Table 3). This is different to results of Zimmermann et al. (2013) who showed highest rate of PIM prescription in the agent group of antidepressants [18]. Since antidepressants and sedatives/hypnotics as most described PIM are commonly given for mental disorders, we examined the proportion of patients with one (or more) related diagnose(s) in our sample. 20.5 % of all patients > 65 years suffered from at least one documented mental disorder (ICD-10, chapter F). Based on this, the differences between our and Zimmermann’s data, concerning the highest rate of PIM prescriptions (as described above), could be explained by the difference in prevalence of depression: whereas 9.4 % of all patients in our sample suffered a documented form of depression, in Zimmermann’s sample there were 12.2 % cases of depression at base line, age-related increasing to > 20 % in the follow-ups [18].

**Table 3:** Top Ten of the prescribed PIM in elderly patients > 65 years (*n* = 1241)

Range	Agent	Total frequency	Total frequency (%)
1	Zopiclone (>3.75 mg/d)	53	4.3
2	Zolpidem (>5 mg/d)	29	2.3
3	Diazepam	24	1.9
	Doxazosin	24	1.9
4	Nifedipine	23	1.9
5	Etoricoxib	18	1.5
6	Medazepam	17	1.4
7	Lorazepam (>2 mg/d)	16	1.3
8	Brotizolam (>0.125 mg/d)	15	1.2
9	Amitryptiline	13	1.1
	Nitrazepam	13	1.1
10	Indomethacin	10	1.0

A significantly higher number of female (F: 27.6 % vs. M: 19.1 %, *χ*^2^ = 12.0/*p* ≤ 0.001) and very old patients (≥80 years: 31.3 % vs. < 80 years: 21.6 %, *χ*^2^ = 11.7/*p* ≤ 0.001) received a PIM prescription. Patients with at least one PIM prescription were characterized by a significantly higher number of chronic diagnoses (M 9.3 ± 3.9 SD) and long-term medication (7.5 ± 3.7 SD) compared to patients without a PIM prescription (8.0 ± 3.6 SD/*t*-test: *p* ≤ 0.001 respectively 5.4 ± 3.0 SD, *t*-test: *p* ≤ 0.001).

### Influencing factors on prescription of PIM

Conducting a logistic regression, we examined the influence of binary associated factors on prescription of PIM in a multivariate model. Including gender, age, documented diagnose of a mental disorder (ICD-10: F), number of diagnoses and number of long-term medication in the regression model, the strongest effect was detected for documented mental disorders (Exp(B) = 2.0/*p* ≤ 0.001), followed by female gender (Exp(B) = 1.6/*p* ≤ 0.01) and number of long-term medication (Exp(B) = 1.2/*p* ≤ 0.001) as predictors for the probability of a PIM prescription. Age and number of diagnoses were not predictive for PIM prescription. The three mentioned predictors explained just 14.9 % of the variance (Nagelkerkes R^2^ = 0.149) of the dependent variable. However, there will be other influencing variables we did not include in the model. Our findings are comparable to other studies that found depression as one of the common mental disorders in elderly patients as main predictor (OR = 2.42) for PIM prescription, followed by the number of prescribed drugs (OR = 1.99) [18]. A positive correlation between the number of prescribed drugs and the risk of PIM in the elderly was also shown in the study of Schubert [19].

### Knowledge and application of PRISCUS-list

PRISCUS was generally known by 5 of 7 interviewed FPs, but the application of the list in daily practice diverged between the interviewees. Several FPs reported its use in daily practice as an instrument for orientation. Others denied or refused to use the list using different arguments pro and contra PRISCUS (see Table 4). Skeptical arguments regarding applicability in daily practice and its benefits for individual patients within the complex context of multimorbidity were reported commonly:

**Table 4:** Pros and Cons of the PRISCUS-list from FPs’ point of view

FPs’ arguments pro PRISCUS	FPs’ arguments contra PRISCUS
•Includes no-go-medication you have to remember every time	Not practicable in FPs’ daily practice since
•Good for orientation	•does often not fit individual patients’ needs
•Possible argument if FP wants to refuse a medication	•within its complex context of multimorbidity
	•does not recommend practicable and pharmacologically based alternatives
	•does not fit experience-based practice
	•does not fit patient demands for a certain PIM based on individual positive experiences
	•limited time in consultations a) to check additional recommendation lists and b) to convince patients for new medication
	•chronological age does not match biological age: lot of patients > 65 years have a younger biological age
	•mistrust in PRISCUS based on missing evidence for medication of multimorbid elderly patients

*Yes, both me and my colleagues have a short version pinned up at our desks. But if you look at the list you get lost even before the consultation with the patient starts. It is not useful at all, unless you want to refuse a prescription of a medication, of course. [FP6]*

The inherent problem of lack of time (the average duration of consultation at FPs’ practices is ca. 8 min per patient in Germany [27]) was mentioned by most of the FPs. This finding is comparable to other studies that also pointed out lack of time as a main barrier for appropriate prescribing [20, 21] or for implementing recommendations of guidelines in daily practice [28].

Several FPs were doubtful regarding the evidence of PRISCUS list since most of pharmacological studies exclude elderly patients, and therefore related evidence is missing. Other studies also showed partly existing skeptical attitudes of physicians regarding cited evidence in guidelines [29]. Two FPs criticized that the PRISCUS list does not recommend *“practicable and pharmacologically based alternatives” [FP6]*. For instance, regarding the prescription of sedatives/hypnotics, FPs reported great uncertainty (see below).

One FP argued that primary care physicians mostly act intuition- and experience-based. From his point of view, evidence-based practice that includes implementation of guidelines in daily practice would not be typical for FPs’ practice. Triangulating these interview data with related data of patient records’ analysis, we detected that this intuition- and experience-oriented FP had a visibly lower prescription rate of PIM while reporting the highest proportion of patients > 65 years. Nevertheless, this single example might not be representative for all FPs. However, intuition-based and evidence-based acting do not exclude each other in the dichotomous way of a “zero-sum relationship” [30].

Social desirability was assumed in some FPs who reported knowing PRISCUS. Asked for their concrete criticisms they first reported weakness of PRISCUS list in general, and, after repeating the question, they apologized for not remembering since they do not use the list. There were two FPs knowing nothing of PRISCUS and PIM at all. This is similar to results of other studies describing educational lacks in primary care as well as hospital physicians, regarding appropriate prescribing in elderly patients [20, 21].

### Reported causes for prescription of potentially inappropriate medication

Most reported cause for PIM prescription was the statement of unavoidability of PIM because having no alternatives in medication, especially in multimorbid patients. FPs prescribing PIM appreciated the related higher risk for unexpected medication-related events and reported to re-consult these patients in shorter periods.

*Regarding polypharmacy, there are no convincing recommendations as to what to prescribe as an alternative. Full stop. That is why I consult these patients (note: with PIM) closely. I will see them several times in the quarter at my practice to keep a close eye on them. [FP2]*

*(Note: As with PIM) you have similar issues with these huge amounts of pain pellets or patients receiving Falithrom (note: Phenprocoumon). Every time something can happen. But this is unavoidable, isn’t it? [FP5]*

Further arguments are related to FPs’ prioritization regarding therapeutic objectives. There are stronger patient-related factors than age that influence their decision of a certain medication. Based on this, the PIM-topic could become less important:

*If you keep the wellbeing of the patient as the primary goal in mind, then a lot will be sorted based on this prioritization. Age seems to be a difficult criterion in general, so patients’ health status and perspectives should be more important. [FP2]*

*Regarding sedatives, benzodiazepine vs. z-drugs it is like being caught between a rock and a hard place. I am certain that it (note: PIM) is not recommended by PRISCUS but there are no alternatives. And the patient needs to get sleep and be pain-free from time to time. So we talk to the patient about the medication, how to limit it time-wise and how to monitor it closely. [FP1]*

Own bad experiences regarding a) change of medication to avoid PIM and b) refusal of following prescription of sedatives/hypnotics (that also belong to PIM if certain dosage is reached) to addicted patients resulted in a kind of resignation.

*You can start making changes in medication, yes, but most of them are not successful. [FP1]*

In context of FPs’ care for patients who are addicted to hypnotics, PIM-topic seemed to become subordinated whereas treatment of addiction got into the main focus:

*Well, you can speak like a priest in the church: if patients have a certain level of addiction it is really difficult. I could refuse them the medication, but then they will go to a different [physician], so this doesn’t make any sense. [FP7]*

This finding is convenient to our previous result of the logistic regression (see above), showing that a documented mental disorder was the strongest predictor for prescription of PIM.

Most of the interviewed FPs described the advantages of caring primarily for patients addicted to hypnotics because getting them under control in “one hand”. But finally they seem to be still perplexed:

*I had special cases of patients, when I said, okay, let’s keep them in “one hand” and see if we can somehow take responsibility for it. Yes, we try to care for them individually. Last time I had a patient, she picked up sleeping pills from her neurologist, from me and from another GP. And then at night she had a bad fall because she was just completely in the swash. Finally she was hospitalized, but how to manage her? I have no magic remedy for this problem. [FP4]*

Most of the FPs described the importance of close cooperation with neurologists/psychiatrists regarding care for patients addicted to hypnotics, but several problems were pointed out. First, they reported patients’ difficulties to get access to a psychiatrist for “just” sleeping disorders and second, they reported communication lacks regarding prescriptions of medications and lacks in referral of clinical information. And third, some of the FPs pointed out an uncritical prescribing by psychiatrists without looking at the patient in their entirety including existing multimorbidity. But especially in the case of out-patient detoxification, FPs underlined the necessity of cooperation. To summarize, most of the FPs saw great challenges and chances in a better cooperation with psychiatrists to improve medical care for (elderly) patients addicted to hypnotics.

## Conclusion

This is to our best knowledge the first published study triangulating PIM prescription data based on the analysis of patients’ records with qualitative interview data regarding case-related motives of PIM prescription. In summary, prescription of PIM was mainly a topic of chronic disease treatment. We find out that it is essential to see FPs in a complex decision making situation with several influencing factors on their prescribing, including:

1. patient-oriented prioritization reflecting i.e. multimorbidity and related polypharmacy, existing mental disorders, gender, patients’ requests [29] and experiences
2. FPs’ experiences in daily practice [29] regarding e.g. (inter) actions of drugs [31], reactions of patients in case of refusal or change of a drug
3. FPs’ knowledge regarding e.g. existing evidence/recommendations and their trust in it
4. dependent on quality of cooperation with in- and out-patient specialist; forwarded information regarding clinical indication and prescribed drugs
5. organizational characteristics, i.e. lack of time in daily practice, follow-up prescriptions of PIM initially prescribed by specialists.

Obviously, there is a problem with the applicability of guidelines and recommendations, rooted in the special characteristics of FPs´ work: the individual consideration of decisions against the background of the knowledge of the patient’s medical history, patient´s characteristics and suspected adherence.

Partly, FPs reported to be not really convinced of their decisions foreseeing associated higher risks of drug related adverse events. Assuming that they will not find medication alternatives for individual multimorbid patients, frequently re-consultations and laboratory tests were seen as methods to detect drug related problems as soon as possible.

The partly detected education lack regarding PIM and PRISCUS shows challenges for further educational tasks that should take the partly missing trust in recommendation and PRISCUS into account even though influence of new guidelines and their recommendations on physicians’ daily practice seems to be limited [29].

## Abbreviations

FP, family practitioner; PIM, potentially inappropriate medication; PRISCUS, PRerequISites for a new health Care model for elderly people with mUltiple morbiditieS

## References

[1] Mangoni AA, Jackson SH (2004). Age-related changes in pharmacokinetics and pharmacodynamics: basic principles and practical applications. Br J Clin Pharmacol.

[2] Beers E, Moerkerken DC, Leufkens HG, Egberts TC, Jansen PA (2014). Participation of older people in preauthorization trials of recently approved medicines. J Am Geriatr Soc.

[3] Cherubini A, Oristrell J, Pla X, Ruggiero C, Ferretti R, Diestre G, Clarfield AM, Crome P, Hertogh C, Lesauskaite V, Prada GI, Szczerbinska K, Topinkova E, Sinclair-Cohen J, Edbrooke D, Mills GH (2011). The persistent exclusion of older patients from ongoing clinical trials regarding heart failure. Arch Intern Med.

[4] Gurwitz JH, Field TS, Harrold LR, Rothschild J, Debellis K, Seger AC, Cadoret C, Fish LS, Garber L, Kelleher M, Bates DW (2003). Incidence and preventability of adverse drug events among older persons in the ambulatory setting. JAMA.

[5] Gurwitz JH, Field TS, Judge J, Rochon P, Harrold LR, Cadoret C, Lee M, White K, LaPrino J, Erramuspe-Mainard J, DeFlorio M, Gavendo L, Auger J, Bates DW (2005). The incidence of adverse drug events in two large academic long-term care facilities. Am J Med.

[6] Reich O, Rosemann T, Rapold R, Blozik E, Senn O (2014). Potentially inappropriate medication use in older patients in Swiss managed care plans: prevalence, determinants and association with hospitalization. PLoS One.

[7] Fialova D, Topinkova E, Gambassi G, Finne-Soveri H, Jonsson PV, Carpenter I, Schroll M, Onder G, Sorbye LW, Wagner C, Reissigova J, Bernabei R (2005). Potentially inappropriate medication use among elderly home care patients in Europe. JAMA.

[8] Beers MH (1997). Explicit criteria for determining potentially inappropriate medication use by the elderly. An Update Arch Intern Med.

[9] Fick DM, Cooper JW, Wade WE, Waller JL, Maclean JR, Beers MH (2003). Updating the Beers criteria for potentially inappropriate medication use in older adults: results of a US consensus panel of experts. Arch Intern Med.

[10] Gallagher P, Ryan C, Byrne S, Kennedy J, O’Mahony D (2008). STOPP (Screening Tool of Older Person’s Prescriptions) and START (Screening Tool to Alert doctors to Right Treatment). Consensus validation. Int J Clin Pharmacol Ther.

[11] Hamilton H, Gallagher P, Ryan C, Byrne S, O’Mahony D (2011). Potentially inappropriate medications defined by STOPP criteria and the risk of adverse drug events in older hospitalized patients. Arch Intern Med.

[12] Laroche ML, Charmes JP, Merle L (2007). Potentially inappropriate medications in the elderly: a French consensus panel list. Eur J Clin Pharmacol.

[13] Mann E, Böhmdorfer B, Frühwald T, Roller-Wirnsberger RE, Dovjak P, Dückelmann-Hofer C, Fischer P, Rabady S, Iglseder B (2012). Potentially inappropriate medication in geriatric patients: the Austrian consensus panel list. Wien Klin Wochenschr.

[14] Rognstad S, Brekke M, Fetveit A, Spigset O, Wyller TB (2009). Straand J+. The Norwegian General Practice (NORGEP) criteria for assessing potentially inappropriate prescriptions to elderly patients: a modified Delphi study. Scand J Prim Health Care.

[15] Holt S, Schmiedl S, Th++rmann PA (2010). Potentially inappropriate medications in the elderly: the PRISCUS list. Deutsches Ärzteblatt Int.

[16] Opondo D, Eslami S, Visscher S, De Rooij SE, Verheij R, Korevaar JC, Abu-Hanna A (2012). Inappropriateness of medication prescriptions to elderly patients in the primary care setting: a systematic review. PLoS One.

[17] Fiss T, Thyrian JR, Fendrich K, Berg N, Hoffmann W (2013). Cognitive impairment in primary ambulatory health care: pharmacotherapy and the use of potentially inappropriate medicine. Int J Geriatric Psychiatry.

[18] Zimmermann T, Kaduszkiewicz H, van den Bussche H, Schön G, Brettschneider C, König HH, Wiese B, Bickel H, Mösch E, Luppa M (2013). Potentially inappropriate medication in elderly primary care patients. A retrospective, longitudinal analysis. Bundesgesundheitsblatt-Gesundheitsforschung-Gesundheitsschutz.

[19] Schubert I, Küpper-Nybelen J, Ihle P, Thürmann P (2013). Prescribing potentially inappropriate medication (PIM) in Germany’s elderly as indicated by the PRISCUS list. An analysis based on regional claims data. Pharmacoepidemiol Drug Saf.

[20] Maio V, Jutkowitz E, Herrera K, Abouzaid S, Negri G, Del Canale S (2011). Appropriate medication prescribing in elderly patients: how knowledgeable are primary care physicians? A survey study in Parma, Italy. J Clin Pharm Ther.

[21] Ramaswamy R, Maio V, Diamond JJ, Talati AR, Hartmann CW, Arenson C, Roehl B (2011). Potentially inappropriate prescribing in elderly: assessing doctor knowledge, confidence and barriers. J Eval Clin Pract.

[22] Hübsch G, Gottschall M, Mergenthal K, Schübel J, Bergmann A, Voigt K (2015). Are Saxonian Family Practices on the Way to Team Practices?. ZFA.

[23] Creswell JW, Fetters MD, Ivankova NV (2004). Designing a mixed methods study in primary care. Ann Fam Med.

[24] Köberlein J, Gottschall M, Czarnecki K, Thomas A, Bergmann A, Voigt K (2013). General practitioners’ views on polypharmacy and its consequences for patient health care. BMC Fam Pract.

[25] Mayring P (2010). Qualitative Inhaltsanalyse.

[26] Münch C. Quality of documentation of diagnoses by GPs with an example of thyroid diseases [Master Thesis at Technische Universität Dresden/Faculty of Medicine] 2015

[27] Van den Brink-Muinen A, Verhaak PF, Bensing JM, Bahrs O, Deveugele M, Gask L, Mead N, Leiva-Fernandez F, Perez A, Messerli V (2003). Communication in general practice: differences between European countries. Fam Pract.

[28] Kastner M, Estey E, Hayden L, Chatterjee A, Grudniewicz A, Graham ID, Bhattacharyya O (2014). The development of a guideline implementability tool (GUIDE-IT): a qualitative study of family physician perspectives. BMC Fam Pract.

[29] Wollny A, Rieger MA, Wilm S (2009). Inadequate reimbursement and the patients themselves may inhibit the implementation of guidelines. Evaluation of the venous leg ulcer guideline of the German Society of Phlebology (DGP) in general and phlebology practices. Z Evid Fortbild Qual Gesundhwes.

[30] Greenhalgh T (2002). Intuition and evidence--uneasy bedfellows?. Br J Gen Pract.

[31] Schubert I, Egen-Lappe V, Heymans L, Ihle P, Feßler J (2009). ‘To Read’ does not Imply ‘To Act upon’: Indicators of the Acceptance of General Practice Guidelines. Results of a Survey among Quality Circles of General Practitioner Centred Care (Hausarztzentrierte Versorgung; HZV). Zeitschrift für Evidenz, Fortbildung und Qualität im Gesundheitswesen.

